# Effectiveness of the ELLA Training for the Promotion of Emotional and Social Competences in Lithuanian Preschool Children

**DOI:** 10.3390/ijerph191912195

**Published:** 2022-09-26

**Authors:** Giedrė Širvinskienė, Dalia Antinienė, Aušra Griciūtė, Liudmila Dulksnienė, Vaidilutė Asisi, Rima Kregždytė, Verena Kerbl, Elfriede Amtmann

**Affiliations:** 1 Department of Health Psychology, Lithuanian University of Health Sciences, Tilžės 18, 47181 Kaunas, Lithuania; 2 Health Research Institute, Lithuanian University of Health Sciences, Tilžės 18, 47181 Kaunas, Lithuania; 3 Department of Languages and Education, Lithuanian University of Health Sciences, Jankaus g. 2, 47181 Kaunas, Lithuania; 4 Autism Therapy Center of the Vkkj, The Sonnwendviertel Outpatient Clinic, Maria Lassnig Street 2, 1100 Vienna, Austria; 5 Department of Preventive Medicine, Lithuanian University of Health Sciences, Tilžės 18, 47181 Kaunas, Lithuania; 6 Department of Education Sciences, Private University College of Teacher Education Augustinum, Lange Gasse 2, 8020 Graz, Austria

**Keywords:** children, preschool, emotional and social competences

## Abstract

By developing the emotional and social competences of children of preschool age, one can expect the prevention of emotional and behavioral problems and a better social and academic adaptation. The aim of this study was to evaluate the effectiveness of the ELLA training for the promotion of emotional and social competences in 3–6-year-old children in preschool education institutions in Lithuania. In total, 140 children aged 3–6 years participated in the quasi-experimental study, of which 86 children were assigned to the experimental group and 54 were assigned to the control group. Children of the experimental group were given a modified program—the ELLA training for the promotion of emotional and social competences. Children’s emotional and social competences were assessed before and after the program. The EMK 3–6 inventory (germ. Inventar zur Erfassung Emotionaler Kompetenzen bei Drei-bis Sechsjährigen, EMK 3–6) was used to conduct a questionnaire survey of teachers and to carry out an individual assessment performed by psychologists in order to assess the children’s competences. The ELLA training significantly improved children’s emotional and social competences. Based on the teachers’ assessment, the children’s self-regulation abilities improved, and based on the children’s individual assessment conducted by psychologists, the application of the program resulted in the improvement of the children’s primary emotions, secondary emotions, and prosocial behavior competences.

## 1. Introduction

### 1.1. Conceptualization of Emotional and Social Competences

Various definitions of emotional and social competences can be found in the scientific literature. Emotional and social competences are often defined as the ability to recognize and regulate emotions, to set and achieve appropriate goals, to make responsible decisions, and to effectively manage interpersonal relationships [1]. Even though there is still no universal definition of emotional and social competences, there is generally an agreement on the areas of competences, i.e., the ability to establish friendships, to communicate with others, to understand and express emotions in acceptable ways, and to resolve conflicts and be empathic [2]. The most favorable time for the development of these competences is childhood, when the formation of connections in the brain is the fastest [3]. When it comes to children, emotional competence, in addition to the aforementioned aspects, also refers to children’s ability to explore the environment and learn [4]. Researchers have found that when children are emotionally competent, their self-evaluation is positive, and they are more resilient in emotionally challenging situations. Conversely, a lack of emotional competence at an early age is associated with a risk of later emotional and behavioral disorders and poor academic achievements [5,6,7]. Usually, children’s emotional and social competence is naturally established in the contexts of family, community, and culture.

Recently, researchers have increasingly emphasized the importance of emotional and social competence in early childhood [8]. Emotional competences acquired during the first years of a person’s life may help predict a child’s later psychological well-being and success [9]. Research has shown that the facilitation of the emotional and social competences of children of preschool and pre-primary age can lead to the children’s better social and academic adaptation. Better emotional and social competences in preschool children are associated with fewer emotional and behavioral disorders [10]. A positive relationship has also been found between young children’s emotional competences and their school readiness and successful peer relationships [11]. Researchers have shown a link between a child’s emotional and social competence in kindergarten and a better education level and fewer psychiatric problems in early adulthood [12].

### 1.2. Possibilities for the Promotion of Emotional and Social Competences at an Early Age

Researchers focus on ensuring that every young child, including those with mild developmental delays or disabilities and those in risk groups, benefits from school-based activities related to the promotion of emotional and social competences [13]. The good news is that research has shown that early intervention using social-emotional competence development strategies can prevent a child’s future behavior problems, antisocial behavior, and many other risk factors [14].

The development of children’s emotional and social competences is greatly influenced by the interaction between parents and children as well as the family environment [15,16]. Parents who are sensitive to their children’s emotional needs tend to raise more emotionally and socially competent children [3]. The importance of the emotional connection between parents and children has been proven in many studies: for example, there is a relationship between the parents’ emotion regulation abilities and the development of these abilities in their children [17]. Various studies have also shown that it is not only the objective emotional relationship between parents and children that is important but also how it is subjectively evaluated by the child at that time or from a time perspective when the child becomes an adult. In other words, a relationship has been found between such indicators of emotional competence as the perception, understanding, and management of emotions and the subjectively perceived parental warmth in childhood [18,19].

Parental involvement in activities carried out at educational institutions is particularly significant in early childhood [20]. Research has shown that parental involvement in the life of an educational institution correlates with children’s academic achievements and prosocial behavior [21]. The parents’ participation in improving children’s emotional and social competences has a similar effect [22]: children must have the opportunity to practice these skills not only in educational institutions but also at home, thus ensuring the applicability of the skills in everyday life.

Teachers also play a very important role in promoting children’s emotional and social competences [23]. With the help of teachers and socialization, children can acquire skills to manage their emotions and express them in an acceptable way. There is evidence that young children understand their own and other peoples’ emotions better when teachers respond to children’s negative emotions in a calm and supportive manner [24,25]. Thus, teachers are an integral part of the development of children’s emotional and social competences; they are the people who can ensure children’s emotional and social well-being. Many preschool teachers are familiar with various aspects of children’s emotional competence, but researchers note that teachers still need specific knowledge, and there is a lack of specialized, effective, and scientifically based programs for the development of emotional and social competence [2,26].

### 1.3. Emotional and Social Competence-Promoting Programs and Their Effectiveness

Recently, a number of emotional and social competence-promoting programs have been created for children up to 5–6 years of age. The programs used for the development of children’s emotional and social competences and the suppression of problematic behavior differ in their content, structure, and scope, which makes their comparison complicated. A significant number of the programs for preschool-age children are preventive and are intended to be applied to the entire kindergarten group, while other programs are specialized—for example, for children with autism spectrum disorders or behavioral and emotional disorders. The programs are tailored to educational institutions or families (parents) and are designed to improve the child’s emotional state and behavior. Examples of programs involving educators working with children in the field of emotional and social education are the following: “Lubo aus dem All!” for preschoolers [27], the Second Step Program ref. [28], the Promoting Alternative Thinking Strategies (PATHS) preventive program [29], Zippy’s Friends developed by the organization Partnership for Children, and others. The effectiveness of these and many other programs has been scientifically proven. Programs for the promotion of emotional competence whose short-term or long-term effectiveness has been confirmed by empirical studies are regarded as scientifically based. For example, the Teaching Pyramid [30], a widely used program based on the Positive Behavior Intervention and Support concept designed to address behavioral difficulties in preschool children aged 3–5 years, was developed to help teachers implement evidence-based practices focused on the emotional and social development of young children. The effectiveness of this program has been verified by its authors, and its biggest advantage is that the emotional competences of all children in the kindergarten group are developed, but the form and intensity of the training varies depending on the individual needs of each child [31].

Another evidence-based program, Fun Friends, was developed for children aged 4 to 6 years [32]. This program is based on the problem-solving theory and cognitive behavioral therapy, its goals being to reduce children’s anxiety in the process of socialization and to teach children emotional skills, coping strategies, and psychological resilience [33]. A study of the effectiveness of this program showed that, after the intervention, children’s anxiety levels decreased, the understanding and recognition of emotions improved, as did the ability to solve conflict situations, and the behavior when dealing with an aggressive person became more effective [34].

One of the empirically based programs is the ELLA training (germ. ELLA, ein Training zur Förderung der emotionalen und sozialen Kompetenz, developed in Austria [35]). The program is named after the main character of the training, ELLA, a hand puppet Giraffe. This program is based on the Model of Affective Social Competence [36] and the Skill-Based Model of Emotional Competence according to Saarni [37]. When evaluating its effectiveness, the authors of the ELLA found an improvement in children’s emotional knowledge, empathy, prosocial behavior, and self-regulation after the intervention. In addition, the evaluation showed that, in the groups that included high-risk children with lower emotional and social competences, knowledge about emotions and empathy improved after the training, the children’s prosocial behavior changed positively, and self-regulation and social competence improved as well [35].

However, the effectiveness of emotional and social competence training programs is still debated [38,39]. The authors of the programs emphasize different positive changes induced by the intervention: they state that children who participated in the programs had better competence in recognizing emotions [34,40], their emotional vocabulary increased, their cooperative behavior, empathy, self-control, and sense of responsibility improved [41], children exhibited lower levels of aggression [42], children had reduced anxiety levels, an improved understanding of emotions, and the ability to deal with conflict situations [27,34], and children also demonstrated an increase in prosocial behavior [43]. A number of systematic reviews and meta-analyses have been devoted to the overall analysis of the effectiveness of emotional and social competence training programs [13,44,45,46,47]. Their results show that intervention programs do strengthen children’s emotional and social competences.

However, even when programs are proven to be effective, it often turns out that their effects are not long-lasting, or the results obtained by researchers are inconsistent. In other words, some researchers document a greater impact from the program, while others report a lesser effect. For these reasons, the effectiveness of programs must be reviewed from time to time, and programs suitable for one cultural environment must be tested before being implemented in another.

### 1.4. The Aim and Significance of the Work

The aim of this study was to evaluate the effectiveness of the modified ELLA training program for the promotion of emotional and social competences in 3–6-year-old children in preschool education institutions of Lithuania. The research hypothesis was that the modified ELLA training for the promotion of emotional and social competences improves the emotional and social competences of 3–6-year-old children. The effectiveness of the training has been evaluated in Austria in a sample of 3–6-year-old children, where improvements in emotional knowledge, empathy/prosocial behavior, and self-regulation competences were found after the training [35]. In other countries, the effectiveness of the training has not yet been evaluated. The study is significant in that, for the first time, the effectiveness of the training was evaluated outside of Austria, in the context of another country and educational system. In terms of the prevalence of emotional and behavioral difficulties in preschool children, Lithuania belongs to the highest-risk group [48], but there is a severe lack of scientifically based intervention programs for preschool children in Lithuania, and, thus, a large number of children of this age who have problems do not receive adequate and timely help. Recently, various emotional and social competence-promoting programs have been implemented in Lithuanian kindergartens, but their effectiveness is not scientifically based. The study was also associated with the development of inclusive education in order to ensure the provision of theoretically based and empirically proven educational models for children with minor or moderate special needs and their parents, teachers, and other specialists. For this reason, the evaluation of the effectiveness of programs for children in order to develop inclusive education and better indicators of children’s mental health is particularly timely and relevant. From 2023, the country will start implementing an inclusive education program in the primary education system; therefore, there is a need for effective and evidence-based emotional and social competence education programs and a special need for such interventions that would be suitable for children with behavioral and emotional difficulties or who fall into risk groups. It is also noteworthy that the training took place during the period of the COVID-19 pandemic. Scientific evidence shows that, during the pandemic, many mental health problems of children such as anxiety, depression, post-traumatic symptoms [49], and emotional and behavioral problems [50] have arisen; thus it is very important to analyze the impact that the training of emotional and social competences during the COVID-19 pandemic has on the development of these competences.

## 2. Materials and Methods

### 2.1. Subjects and Study Procedures

During the study, the effectiveness of the ELLA training for the promotion of emotional and social competences in 3–6-year-old children was evaluated. The quasi-experimental study was carried out in seven preschool and pre-primary education institutions in four different counties of Lithuania in 2022 (Kaunas, Vilnius, Klaipėda district, and Marijampolė). In the institutions participating in the study, 1–2 groups of kindergarten children were randomly selected, from which the experimental group was formed, and another 1–2 other groups of kindergarten children were selected, from which the control group was formed. Parents of children who attended randomly selected kindergarten groups and met the inclusion criteria were informed about the study, invited to participate in the study, and asked to sign the informed consent form. The inclusion criterion for the children participating was that the child was 3–6 years old. There was also an intention to form experimental and control groups similar in terms of gender, age proportions, and the number of children in each group. The children’s teachers filled out questionnaires about the children, and socio-demographic data were collected from one of the parents.

The emotional and social competences of the children in the experimental and control groups were assessed twice. The first evaluation of the children was carried out before starting the ELLA training for the promotion of emotional and social competences, and the second was carried out after the completion of the training. The children of the experimental group participated in the modified ELLA training; based on the training methodology, separate groups of 3–4-year-old and 5–6-year-old children were formed, each consisting of 8–10 children [35]. A total of eight groups were formed. The training was conducted by trained psychologists together with kindergarten teachers, who assisted them and also participated in special training. The children of the control group were not exposed to the training and had no contact with the children of the experimental group during the study. To evaluate the effectiveness of the training, information was obtained from different informants—psychologists who performed individual assessments of the children and teachers working with children in the kindergarten who filled out questionnaires, which allowed for controlling for possible methodological bias [51]. The teachers were given a questionnaire using the EMK screening, and the psychologists performed an individual assessment of the children’s emotional and social competences using the EMK 3–6. Additionally, to avoid the bias effect, the intervention training for children was carried out and evaluated by different researchers.

The parents of the children of the experimental group were introduced to the main ideas and principles of the program and were encouraged to observe and reinforce the behavior of their children as they expressed their emotional and social competences. During the ELLA training, children’s emotional and social competences were developed with the help of various tasks twice a week for about 30–60 min a day. In total, eight sessions were completed in about 4–5 weeks. The Lithuanian version of the ELLA training was prepared for the study. The authors of the program, E. Amtmann and V. Kerbl, trained the researchers (psychologists and pedagogues) on how to conduct the training and provided supervision during its implementation in order to ensure the standardized conduction of educational programs.

In total, 143 children were selected for the experimental hroup or the control group. Of these, 66 (47.1%) were boys, and 74 (52.9%) were girls. There were 86 (61.4%) children aged 3–4 years and 54 (38.6%) children aged 5–6 years. A total of 89 children were selected for the experimental group, and 54 were assigned to the control group. For some reason, three children did not start attending the training and were excluded from further analysis. Thus, a total of 86 children (the experimental group) participated in the ELLA training.

### 2.2. Intervention Program

The dependent variables of the study were changes in children’s emotional and social competences before and after the ELLA training, and the independent variable of the study was the educational intervention program—the ELLA training for the promotion of emotional and social competences (germ. ELLA, ein Training zur Förderung der emotionalen und sozialen Kompetenz) [35], intended for kindergarten teachers who want to carry out primary preventive work and provide assistance to children aged 3 to 6. The goal of the training is the development of emotional and social competences, based on C. Saarni’s concept of basic emotional competences [37], the Affective Social Competences Model [36], and the Pyramid Model [5]. Four universal emotional competences fall within the domain of capacity building: awareness of emotions, understanding of emotions, empathy, emotional regulation, and basic social skills.

Ella the Giraffe hand puppet is used as the main character in the training, as well as other teaching material. The tasks are presented to children in an appealing way: a game that entails music, role playing, various discussions, stories for relaxation and exercises, storytelling, and group games.

During the study, a modified and Lithuanian-translated version of the training program was used. The training was carried out more intensively—twice a week instead of once a week; thus, it was compressed into 4–5 weeks. The training was conducted by two trained specialists—a psychologist and a kindergarten educator—instead of one teacher, as in the original version. The Lithuanian version of the program song was created and used. The modified program consisted of four stages (a total of eight sessions):

(1) Awareness and Expression of Emotions (three sessions): during the sessions, children talk with the specialists in the group about the basic emotions of anger, sadness, joy, fear, and boredom and/or interest and the ways in which they can be expressed. Children are encouraged to reflect on their feelings and state and express them accordingly, and they are taught to recognize and name the main emotions. Emotion cards are used during sessions.
(2) Understanding and Emotion Knowledge (three sessions): various types of expressions of emotions are discussed; children are taught to observe the expression of each other’s emotions during a play, to recognize them, and to imitate them. The individual expression of emotions and their causes and consequences are discussed. Emotions are expressed during play; emotion cards are used to facilitate the discussions.
(3) Empathy and Prosocial Behavior (one session): based on basic emotions and related personal experiences, children are encouraged to empathize with another person and share their experiences in the group. Thinking about what another person might need when he or she is upset, sad, or afraid can lead to empathy and prosocial behavior.
(4) Emotional Regulation (one session): real-life stories are discussed, based on visual aids, to show what happens in the human body when a person is angry. In a group discussion, various emotional regulation strategies are discussed and tested. The creative use of various emotional regulation techniques is encouraged, and hand puppets with different emotional expressions are used.

The theoretical background of the program, descriptions of the sessions, and the necessary teaching material are presented in the book of the program authors [35].

### 2.3. Instrument and Variables 

To evaluate the emotional and social competences of the children, we used the Inventory to Survey Emotional Competences for Three- to Six-Year-Olds (EMK 3–6) (germ. Inventar zur Erfassung emotionaler Kompetenzen bei Drei- bis Sechsjährigen, EMK 3–6) [52]. The EMK 3–6 allows for a comprehensive evaluation of the emotional and social competences of children aged 3 to 6.5 years. The instrument consists of two parts: EMK screening and EMK 3–6. The instrument was translated from German into Lithuanian by the authors of the article.

EMK screening: This is a questionnaire consisting of three scales: Emotion Knowledge (four statements, scores 0–12), Empathy (eight statements, scores 0–24), and Self-Regulation (five statements, scores 0–15). Examples of statements are: The child expresses his or her feelings properly with his facial expressions; He or she praises other children when they do something good; If a child has to wait, he or she can do something on his or her own (e.g., play or sing). The internal consistency of the subscales in the individual subscales ranges from 0.90 to 0.94, as reported in the test manual. In the study sample, the Cronbach alpha indicators of the internal consistency of the scales were as follows: Emotion Knowledge scales, 0.64; Empathy scales, 0.92; and Self-Regulation scales, 0.77. During the study, the EMK screening questionnaire was filled out by the teachers.

EMK 3–6: In this study, we used three subscales that test the domains of Emotion Knowledge, Emotional Regulation, and Empathy: Primary Emotions (19 items), Secondary Emotions (14 items), and Prosocial Behavior (21 items). In the Primary Emotions and Secondary Emotions subscales, the tasks were aimed at recognizing and naming emotions, naming the facial expressions of emotions, and naming the causes of emotions as well as emotional regulation strategies. The subscale Prosocial Behavior includes such competences as the ability to spontaneously express prosocial behavior, recognize prosocial decisions, and justify them. Children’s competences are assessed by using a book of stimuli, wooden figures, and various tasks presented to the child. In the test manual, the internal consistency (Cronbach’s alpha) of individual EMK 3–6 subscales ranges from 0.78 to 0.90 [52]. In this study, the following indicators of internal consistency were obtained: Primary Emotions, 0.79; Secondary Emotions, 0.72; and Prosocial Behavior, 0.89, which can be regarded as good.

### 2.4. Statistical Analysis

Descriptive, comparative, and relational analysis methods were applied in the statistical analysis of the data. Means, standard deviations, medians, and percentiles (quartiles and terciles) were calculated for the description of quantitative data. The correspondence of the distributions with the normal distribution was checked by applying the Kolmogorov–Smirnov test. Frequencies were calculated for the description of categorical data.

The Chi-square test was used for the comparison of categorical variables between independent groups. Depending on the sample size, the exact (for small samples) and the asymptotic χ^2^ tests were used. The Mann–Whitney *U* test was used to compare quantitative variables between two independent groups. Changes in quantitative values were evaluated using the Wilcoxon signed rank test. The evaluation of the effectiveness of the ELLA training was performed by means of a comparison of the experimental and control groups. Comparative statistical methods allowed for the evaluation of the differences between groups and changes within groups. Differences and relationships were regarded as statistically significant when *p* < 0.05. IBM SPSS Statistics 27 (IBM Corp., Armonk, NY, USA) software was used for the statistical analysis.

### 2.5. Ethical Statement

The study was conducted with the permission of the Kaunas Regional Biomedical Research Ethics Committee No. BE-2-73.

## 3. Results

### 3.1. Indicators of Children’s Emotional and Social Competences

The study showed that, according to the results of the EMK screening (teachers’ questionnaire) of the entire sample of children (the experimental and the control groups), the mean score on the Emotion Knowledge scale was 8.63 (SD = 2.76), on the Empathy scale, it was 16.05 (SD = 6.18), and on the Self-Regulation scale, it was 11.33 (SD = 3.31). The comparison of the mean scores of the scales in the age groups showed that the children’s emotional and social competences did not differ statistically significantly between the age groups; the Mann–Whitney *U* test used to evaluate the difference yielded *p* > 0.05 (Table 1).

We also compared the competences between the genders, but the comparison showed that there was no difference in the emotional and social competences between girls and boys, as assessed by the teachers; the Mann–Whitney *U* test applied for the evaluation of the difference showed *p* > 0.05 (Table 2).

The psychologists carried out individual assessments of the children using the EMK 3–6, obtaining the results of the children’s emotional and social competences. Concerning the children’s age, the evaluation showed that, in the group of 5–6-year-old children, the values of the T-scores of Primary Emotions were higher compared to the respective values in 3–4-year-old children, while the values of the T-scores of Secondary Emotions were found to be higher in the group of younger children (Table 3). The Mann–Whitney *U* test used for the evaluation of the difference yielded *p* < 0.06 for these two competences.

Gender differences were also evaluated, and the results showed that girls’ competences of Primary Emotions were higher than boys’, while the competences of Secondary Emotions and Prosocial Behavior did not differ statistically significantly between the genders; *p* > 0.05 was yielded by the Mann–Whitney *U* test used to evaluate the difference (Table 4).

### 3.2. Comparison of Experimental and Control Group Subjects According to Gender, Age, and Parental Education

The study aimed for an equal distribution of children by gender and age within the experimental and control groups. Before the experiment, the subjects of the experimental and control groups were compared in terms of gender and age, as well as parental education, and the comparison was performed using the Chi-square test. The experimental and control groups did not differ in terms of age (χ^2^ = 0.087, *p* = 0.859), gender (χ^2^ = 0.288, *p* = 0.606), or parental education (χ^2^ = 4.424, *p* = 0.231) (Table 5).

### 3.3. Comparison of the Experimental and Control Group Subjects According to Estimates of Emotional and Social Competences

Before the experiment, using the results of the EMK screening for teachers, the similarities of the structure between the experimental and control groups according to individual subscales were checked. As can be seen, the differences in the estimates of the EMK screening subscales of the first assessment between the experimental and control groups were not statistically significant (Table 6). The Mann–Whitney *U* test used to evaluate the differences yielded *p* > 0.05.

Before the experiment, we compared the T-scores of the first assessment in the subscales of the EMK 3–6 obtained in the experimental and control groups (Table 7). During the first assessment, the differences in the T-scores of Primary Emotions and Prosocial Behavior in the EMK 3–6 between the experimental and the control groups were statistically significant, being higher in the experimental group than in the control group. The Mann–Whitney *U* test used to evaluate the differences yielded *p* < 0.05. Meanwhile, the difference in the T-scores of the Secondary Emotions in the EMK 3–6 between the experimental group and the control group was not statistically significant during the first assessment. The Mann–Whitney *U* test used for the evaluation of the difference yielded *p* > 0.05.

### 3.4. Changes in the Results of the First (Before the Experiment) and Second (After the Experiment) Assessments of the EMK Screening and the EMK 3–6 Data in the Experimental and Control Groups

Based on the EMK screening data, changes in subscale estimates were determined in the experimental and control groups (Table 8).

In the control group, changes in all subscale estimates were not statistically significant. The Wilcoxon signed rank test used to evaluate the changes yielded *p* > 0.05. A statistically significant increase in the estimate in the experimental group was obtained in the Self-Regulation subscale; *p* = 0.04 was yielded by the Wilcoxon signed rank test used for the assessment of changes. In the Emotion Knowledge subscale, a trend of a change was also observed, although the change was not statistically significant. Thus, children’s self-regulation improved after the training.

The changes in the subscale estimates of the EMK screening questionnaire of the experimental group were evaluated according to the duration of the children’s participation in the ELLA training (the number of sessions). The children were distributed into two groups: in the first group, the children participated in one to five sessions, and in the second group, the children participated in six to eight sessions (Table 9).

In the group of children who attended more sessions (6–8), a statistically significant increase in the estimate was found in only the Emotion Knowledge subscale, but a trend of change was also observed in the Self-Regulation subscale. The Wilcoxon signed rank criterion was used to evaluate the changes: in the first case, *p* = 0.014 was yielded, and in the second case, *p* = 0.066 was yielded.

In the study, we evaluated changes in the T-scores between the first and second assessments of children’s emotional competence using the EMK 3–6. We compared the changes in the indicators separately for the experimental and control groups (Table 10).

In the experimental group, a statistically significant increase in the T-score of the EMK 3–6 subscales of Primary Emotions, Secondary Emotions, and Prosocial Behavior was found. The Wilcoxon signed rank test used for the assessment of changes yielded *p* < 0.001.

A statistically significant increase in the T-score of the Primary Emotions subscale of the EMK 3–6 was also found in the control group. The Wilcoxon signed rank test used for the assessment of changes yielded *p* = 0.043. Changes in the T-scores of the Secondary Emotions and Prosocial Behavior subscales were not statistically significant. It is important to note that, when comparing the averages, the changes in the experimental group were greater and statistically more significant than those in the control group.

In the experimental group, changes in subscale scores were evaluated according to the number of sessions of the ELLA training the children participated in (the children participated in either one to five sessions or six to eight sessions). Groups of children who attended one to five sessions and those who attended six to eight sessions were compared (Table 11).

The increase in the T-score of the EMK 3–6 subscales of Primary Emotions, Secondary Emotions, and Prosocial Behavior was statistically significant in both groups formed according to the number of sessions attended. The change was statistically more significant in the group of children who attended more sessions. The Wilcoxon signed rank test showed *p* < 0.05.

## 4. Discussion

It is known that children’s language and executive functions develop most rapidly at the age of 3–6 years; thus, this is the most favorable time to develop their emotional and social competences [47]. The development of these competences is so important that it could be included in educational programs alongside the development of other academic abilities [53].

From the point of view of developmental psychology, the formation of emotional and social competences is affected by the implementation/presence of intervention programs and their nature, but other factors are also important, including the child’s age and gender [54]. Researchers of emotional and social competence are well aware (and it has been empirically proven) that, in childhood, emotional and social skill scores in girls are higher than in boys [55,56]. One of the many possible explanations is that girls’ self-regulation skills, as a certain aspect of the executive function, are more developed [57]. It has also been established that, as a child grows and his or her mental maturity increases, so do his or her emotional abilities—the understanding of emotions and emotional regulation [58]. Some researchers claim that the recognition and naming of emotions improves from the age of 3 to 4–5 years, and this development slows down after the age of 5 [59]. Due to the aforementioned irregularities, in our study, the data of the experimental group were compared with those of the control group to make both groups similar in various aspects before the intervention. Therefore, these factors (i.e., age and gender) were taken into account when forming the experimental and control groups. The analysis of the data showed that the differences in the gender and age of the children selected for the study were insignificant.

For a similar reason, we checked the equivalence of the groups in terms of emotional and social competences before the experiment and found that the results of the experimental group were better than those of the control group in the Primary Emotions and Prosocial Behavior subscales of the EMK 3–6. Thus, similar to the authors of the ELLA training, the authors of this study also had a problem of inconsistency of the primary data between the exposure and the control groups before the application of the educational program [35,60]. Poorer pre-exposure performance of the control group predicts stronger effects of the program for this group of subjects [43]. On the other hand, based on the results of the EMK screening in the experimental and control groups before the experiment, no statistically significant differences were found between the groups. Thus, in summary, it can be said that the starting positions of both the experimental and control group subjects were quite similar overall.

It is important to mention that the assessment of children’s emotional and social competences was based on information obtained from two different sources—a questionnaire survey of teachers based on the EMK screening and an individual examination of the children conducted by psychologists using the EMK 3–6. This ensured greater objectivity of the data. It has been established that cross-informants (in this case, teachers) are especially important in the psychological assessment of a child because the momentary assessment of a child often depends on the context [61], and, when assessing children’s emotional and social competences, the compatibility of the informants often ranges from low to moderate [62]. Therefore, ensuring the presence of several sources of information in child assessment has become the “gold standard” [61].

During the evaluation of the effectiveness of the ELLA training from the teachers’ point of view, the EMK screening data showed that, in the experimental group, a statistically significant increase in the score was only observed in the Self-Regulation subscale, while no changes were detected in the control group. A quasi-experimental study by J. Mihic and her colleagues also proved that competence development improves children’s self-regulation [63]. Similar results were obtained by many other researchers [64,65]. A slight trend of change was also observed in the Emotion Knowledge subscale. Changes in Emotion Knowledge after interventions have been recorded by other researchers as well [40,66,67]. When evaluating the results of the EMK screening according to the number of the training sessions attended, in the experimental group of children who attended more sessions (6–8), an increase in the score was found in the Emotion Knowledge subscale, and a slight increasing trend was observed in the Self-Regulation subscale. There were no statistically significant changes in the groups of children who attended fewer than six sessions. The comparison of the results of this study with those obtained by the authors of the ELLA training program by testing the effectiveness of the program in Austria in 2017–2018 showed that large differences in Emotion Knowledge, Empathy, and Self-Regulation can be seen in different groups. In these areas, teachers rated the children of the experimental group much better than they rated the children of the control group [35].

The assessment of the changes in the children’s evaluation results obtained with the help of the EMK 3–6 after the educational training showed that, in the experimental group, the indicators of the Primary Emotions, Secondary Emotions, and Prosocial Behavior subscales increased—not by much, but statistically significantly. That the effects of emotional and social competence training programs are not large is a relatively common finding [23]. In addition, it should be noted that the educational program was relatively short, i.e., 4–5 weeks, while, in order to form stable and long-term skills, months or even years of training are needed [68]. It is also logical that, as the scores of the Primary and Secondary Emotions increased, the Prosocial Behavior indicators increased as well: it is natural that, when children’s emotional abilities improve, their interpersonal behavior becomes more effective, and the number of the forms of prosocial behavior increases [69].

The comparison of the results according to the number of sessions attended showed that the indicators of emotional competence increased statistically significantly both in children who attended one to five sessions and in those who attended six to eight sessions. It should be noted that, in general, the opinions of researchers regarding how long emotional and social competence-promoting programs should be carried out for in order to achieve an effect differ: for example, according to Moore and his colleagues, the effect of the program is stronger if the duration of the program is longer [70], but other researchers believe that it is not the duration of the intervention that has a greater impact on emotional and social competences but rather the content of the program and its professional application [71].

In summary, it can be noted that the results of the ELLA training obtained with the help of the EMK 3–6 were significantly better than those obtained via the EMK screening. This may mean that teachers did not assess the changes in children’s emotional and social competences objectively enough, which indicates the need to use several sources of information in this kind of research, such as a detailed psychological examination of the child, and the involvement of parents in the evaluation of changes in emotional and social competences could also be significant.

It can be assumed that this training will be effective not only for Lithuanian and Austrian children but also in other contexts of Western cultures. 

It is still a matter of discussion how national or ethnical backgrounds can influence the possibilities of programs that develop emotional and social competences [72]. It is thought, however, that cultural relativity would influence the attitudes towards emotional-social competencies: the importance of some emotional competencies is stressed more, while the importance of others is downplayed due values that exist in society. In order to integrate ELLA practices in cultures different from Western cultures, i.e., more collectivist societies, it should be revised according to a specific culture’s needs. Despite that, it would be useful to look into the efficacy of the program in such cultures.

To date, the effectiveness of the training has not been tested in samples of children with neurodevelopmental problems such as autism. However, considering the developmental perspective, developmentally delayed children could also benefit from the ELLA training. Any kind of developmental delay usually entails a lack of social-emotional skills. Children with the autism spectrum disorder have difficulties getting involved in social relationships and regulating emotions. It can be assumed that certain content could (and should) be adapted for autistic children considering the severity of symptoms and the functional level of the child according to the ICD-11 criteria [73]. 

The evidence-based ELLA training is very necessary for Lithuanian preschool education, as it will allow teachers to reliably develop the emotional and social abilities of children.

### Strengths and Limitations

Several strengths of the study are worth mentioning. One of the strengths is that the assessment of children’s emotional and social competences was based on several sources of information, i.e., a questionnaire survey of the teachers as well as an individual evaluation of the children’s competences by psychologists using the EMF 3–6. When the children were presented with various tasks that they had to solve, they had to answer the questions presented and evaluate the situations. The inclusion of several informants increased the objectivity of the research data. Another strength of the study was that the researchers were trained in the application of the training program and evaluation instruments and also received consultations along the way, which allowed for a high-quality implementation of the training program and application of the evaluation instruments. The researchers received supervision from the authors of the training program, and consultations on the use of the evaluation instruments were received from a researcher with many years of experience. Another strength of the study is that the research has become an empirical basis for the development of emotional and social competences in preschool children.

The study also had several limitations.

First, only the short-term effects of the training program on children’s emotional and social competences were evaluated, assessing changes in competences immediately after the completion of the training. It is well known that the consolidation of emotional and social competences in young children requires a longer duration of the intervention [47].

Second, the study and the training program took place during the COVID-19 pandemic, which complicated the conditions for conducting the study and the training program, making it more difficult to organize the study and the program. The pandemic put stress on researchers, and the risk of lower child attendance increased.

In future research, it would be important to carry out the training program for a longer period of time, with sessions held once a week, and also to evaluate the long-term effects of the program by performing a repeated measurement several months after the completion of the program. In addition, the study showed the importance of using several sources of information in the evaluation of children’s emotional and social competences, not limiting the evaluation to questionnaire surveys of adults but also applying a detailed examination of children’s competences.

Future studies may be directed towards the application of ELLA practices and the application towards children with special needs. It is important to find out how the development of emotional and social competencies may affect a child’s other capabilities such as attention. We hope that by developing the knowledge, understanding, and expression of emotions, attention is also developed, as children are taught to concentrate on, observe, and hear what they are being told by others. Moreover, it is important to analyze the expedience of the participation of parents in the realization of the program and the influence that socio-demographic factors may have on the results of children’s emotional competencies. 

## 5. Conclusions

The study revealed that even a relatively short emotional and social competence-promoting program, when carried out professionally, can be effective. The research hypothesis was confirmed: the ELLA Training for the Promotion of Emotional and Social Competences significantly improved children’s emotional and social competences. Based on the teachers’ assessment, the children’s emotional regulation abilities improved, and based on the children’s individual assessments conducted by psychologists, the training also improved the children’s primary emotions, secondary emotions, and prosocial behavior competences.

## Figures and Tables

**Table 1:** Results of the distribution of the EMK screening scores in the age groups.

Scale	3–4 Years		5–6 Years		*p*
	*n*	Mean Score (SD)	*n*	Mean Score (SD)	
Emotion Knowledge	86	8.27 (2.86)	54	9.19 (2.61)	0.065
Empathy	86	15.33 (6.68)	54	16.98 (5.48)	0.213
Self-Regulation	86	11.19 (3.46)	54	11.68 (3.24)	0.404

**Table 2:** Results of the distribution of the EMK screening scores among girls and boys.

Scale	Girls		Boys		*p*
	*n*	Mean Score (SD)	*n*	Mean Score (SD)	
Emotion Knowledge	74	8.95 (2.70)	66	8.26 (2.87)	0.123
Empathy	74	16.55 (6.20)	66	15.30 (6.35)	0.205
Self-Regulation	74	11.86 (3.20)	66	10.83 (3.50)	0.051

**Table 3:** Results of the distribution of the scores of the EMK 3–6 scales (emotional and social competences) in the age groups.

Scale	3–4 Years		5–6 Years		*p*
	*n*	Mean T-Scores (SD)	*n*	Mean T-Scores (SD)	
Primary emotions	86	52.19 (10.43)	54	56.35 (12.19)	0.029
Secondary emotions	86	56.14 (51.43)	54	51.43 (8.60)	0.003
Prosocial behavior	86	51.01 (11.80)	54	51.63 (10.67)	0.722

**Table 4:** Results of the distribution of the scores of the EMK 3–6 scales (emotional and social competences) in boys and girls.

Scale	Girls		Boys		*p*
	*n*	Mean T-Scores (SD)	*n*	Mean T-Scores (SD)	
Primary emotions	74	55.93 (10.94)	66	51.39 (11.27)	0.017
Secondary emotions	74	53.58 (9.14)	66	55.15 (9.70)	0.169
Prosocial behavior	74	51.26 (9.80)	66	51.24 (12.93)	0.856

**Table 5:** Distribution of children in the experimental and control groups by gender, age, and parental education.

Groups			Experimental Group *n* (%)	Control Group *n* (%)	Total *n* (%)
Girls	Age	3–4 years	27 (57.5)	15 (55.6)	42 (56.8)
		5–6 years	20 (42.6)	12 (44.4)	32 (43.2)
	Total		47 (100)	27 (100)	74 (100)
Boys	Age	3–4 years	25 (64.1)	19 (70.4)	44 (66.7)
		5–6 years	14 (35.9)	8 (29.6)	22 (33.3)
	Total		39 (100)	27 (100)	66 (100)
Total	Age	3–4 years	52 (60.5)	34 (63.0)	86 (61.4)
		5–6 years	34 (39.5)	20 (37.0)	54 (38.6)
	Total		86 (100)	54 (100)	140 (100)
Parental education	Secondary or lower		7 (8.1)	5 (9.3)	12 (8.6)
	Vocational		8 (9.3)	10 (18.5)	18 (12.9)
	Higher (non-university)		21 (24.4)	7 (13.0)	28 (20.0)
	Higher (university)		21 (58.1)	32 (59.3)	82 (8.6)
	Total		86 (100)	54 (100)	140 (100)

**Table 6:** Comparison of the experimental and control groups according to the EMK screening scale estimates before the ELLA training.

Scale	Experimental Group				Control Group				*p*
	Mean	SD	Median	Q1–Q3	Mean	SD	Median	Q1–Q3	
Emotion Knowledge	8.63	2.74	9.00	7.00–11.00	8.62	2.91	9.00	7.50–11.00	0.950
Empathy	15.70	6.46	17.00	11.00–21.00	16.40	6.16	18.00	14.00–21.50	0.606
Self-Regulation	11.45	3.12	12.00	9.00–14.00	11.34	3.77	13.00	9.00–14.00	0.834

**Table 7:** Comparison of the EMK 3–6 results of the first assessment in the experimental and control groups.

Scale	Experimental Group				Control Group				*p*
	Mean	SD	Median	Q1–Q3	Mean	SD	Median	Q1–Q3	
Primary emotions	56.28	9.51	57.00	50.00–63.25	50.00	12.13	48.00	38.00–63.50	0.016
Secondary emotions	54.38	8.77	51.00	50.00–59.25	54.57	10.27	51.00	46.00–60.00	0.981
Prosocial behavior	53.29	10.63	55.50	44.00–61.25	47.94	11.59	46.00	38.00–57.50	0.009

**Table 8:** Changes in the EMK screening results when comparing the first and second assessment data in the experimental and control groups.

	First Assessment				Second Assessment				*p*
	Mean	SD	Median	Q1–Q3	Mean	SD	Median	Q1–Q3	
Emotion Knowledge									
Experimental group	8.63	2.74	9.00	7.00–11.00	9.16	2.28	10.00	7.00–11.00	0.058
Control group	8.62	2.91	9.00	7.50–11.00	8.70	2.50	9.00	7.00–11.00	0.861
Empathy									
Experimental group	15.70	6.46	17.00	11.00–21.00	16.57	5.29	17.00	13.00–21.00	0.242
Control group	16.40	6.16	18.00	14.00–21.50	16.02	6.76	18.00	12.50–21.50	0.540
Self-Regulation									
Experimental group	11.45	3.12	12.00	9.00–14.00	12.22	2.31	13.00	11.00–14.00	0.040
Control group	11.34	3.77	13.00	9.00–14.00	11.06	4.15	13.00	9.50–14.00	0.814

**Table 9:** Changes in the EMK screening between the data of the first and second assessments in the experimental group according to the number of sessions attended.

	First Assessment				Second Assessment				*p*
	Mean	SD	Median	Q1–Q3	Mean	SD	Median	Q1–Q3	
Emotion Knowledge									
1–5 sessions	9.00	2.92	9.00	7.50–11.50	8.76	2.22	9.00	7.50–11.00	0.696
6–8 sessions	8.53	2.70	9.00	7.00–11.00	9.26	2.30	10.00	7.00–11.00	0.014
Empathy									
1–5 sessions	15.94	6.51	19.00	8.00–20.50	17.29	4.91	19.00	13.00–21.00	0.532
6–8 sessions	15.64	6.45	16.50	11.75–22.00	16.38	5.40	16.00	13.00–21.00	0.320
Self-regulation									
1–5 sessions	11.88	3.33	13.00	10.50–14.00	12.94	1.78	14.00	11.50–14.00	0.360
6–8 sessions	11.33	3.08	12.00	9.00–14.00	12.03	2.40	12.00	11.00–14.00	0.066

**Table 10:** Changes in the results of the EMK 3–6 in the experimental and control groups, comparing the data of the first and second assessments.

	First Assessment				Second Assessment				*p*
	Mean	SD	Median	Q1–Q3	Mean	SD	Median	Q1–Q3	
Primary emotions									
Experimental group	56.28	9.51	57.00	50.00–63.25	62.99	8.14	64.00	57.00–68.25	<0.001
Control group	50.00	12.13	48.00	38.00–63.50	52.59	11.58	55.00	44.00–69.00	0.043
Secondary emotions									
Experimental group	54.38	8.77	51.00	50.00–59.25	59.45	10.33	58.00	51.00–66.00	<0.001
Control group	54.57	10.27	51.00	46.00–60.00	55.59	9.35	52.00	47.00–61.00	0.270
Prosocial behavior									
Experimental group	53.29	10.63	55.50	44.00–61.25	59.74	9.80	61.00	54.00–66.25	<0.001
Control group	47.94	11.59	46.00	38.00–57.50	50.51	11.45	50.00	40.00–67.00	0.051

**Table 11:** Changes in the results of the EMK 3–6 in separate subscales, comparing the data of the first and second assessments in the experimental group, which were formed according to the number of sessions attended.

	First Assessment				Second Assessment				*p*
	Mean	SD	Median	Q1–Q3	Mean	SD	Median	Q1–Q3	
Primary emotions									
1–5 sessions	56.77	9.52	60.00	50.00–63.00	63.77	8.62	62.00	56.00–69.50	0.004
6–8 sessions	56.18	9.58	57.00	50.00–63.50	62.83	8.10	64.00	57.50–68.50	<0.001
Secondary emotions									
1–5 sessions	52.38	9.09	50.00	48.00–56.00	59.85	12.31	56.00	51.00–70.50	0.016
6–8 sessions	54.78	8.72	52.00	50.00–60.00	59.37	9.99	59.00	51.00–66.00	<0.001
Prosocial behavior									
1–5 sessions	52.54	9.62	54.00	44.50–61.50	59.54	12.33	62.00	51.00–70.00	0.012
6–8 sessions	53.45	10.88	56.00	44.00–61.50	59.78	9.32	61.00	55.00–65.50	<0.001

## Data Availability

Not applicable.
